# Does the global activity limitation indicator measure participation restriction? Data from the European Health and Social Integration Survey in Spain

**DOI:** 10.1007/s11136-021-03057-z

**Published:** 2021-12-09

**Authors:** Julio Cabrero-García, Juan Ramón Rico-Juan, Antonio Oliver-Roig

**Affiliations:** 1 Department of Nursing, University of Alicante, Carretera San Vicente del Raspeig s/n, 03690 San Vicente del Raspeig-Alicante, Spain; 2 Department of Software and Computing Systems, University of Alicante, Carretera San Vicente del Raspeig s/n, 03690 San Vicente del Raspeig-Alicante, Spain; 3 Department of Nursing, University of Alicante, Carretera San Vicente del Raspeig s/n, 03690 San Vicente del Raspeig-Alicante, Spain

**Keywords:** Global activity limitation indicator, Self-rated health, Participation restriction, Differential item functioning, Relative importance, Validity

## Abstract

**Purpose:**

The global activity limitation indicator (GALI) is the only internationally agreed and harmonised participation restriction measure. We examine if GALI, as intended, is a reflective measure of the domains of participation; furthermore, we determine the relative importance of these domains. Also, we investigated the consistency of response to GALI by age and gender and compared the performance of GALI with that of self-rated health (SRH).

**Methods:**

We used Spanish data from the European Health and Social Integration Survey and selected adults aged 18 and over (*N* = 13,568). Data analysis, based on logistic regression models and Shapley value decomposition, were also stratified by age. The predictors of the models were demographic variables and restrictions in participation domains: studies, work, mobility, leisure and social activities, domestic life, and self-care. The GALI and SRH were the response variables.

**Results:**

GALI was strongly associated with all participation domains (e.g. for domestic life, adjusted OR 24.34 (95% CI 18.53–31.97) in adult under 65) and performed differentially with age (e.g. for domestic life, adjusted OR 13.33 (95% CI 10.42–17.03) in adults over 64), but not with gender. The relative importance of domains varied with age (e.g. work was the most important domain for younger and domestic life for older adults). The results with SRH were parallel to those of GALI, but the association of SRH with participation domains was lowest.

**Conclusions:**

GALI reflects well restrictions in multiple participation domains and performs differently with age, probably because older people lower their standard of good functioning.

**Supplementary Information:**

The online version contains supplementary material available at 10.1007/s11136-021-03057-z.

## Plain English summary

Global health indicators based on survey data allow monitoring of population health. The most critical measure is based on restrictions in social participation (i.e. restrictions in performing social roles and activities such as home life, leisure, work, and so on) for health reasons. Precisely, the great diversity of aspects of participation, together with the unequal relevance of these for different age groups, cultures, etc., makes it very difficult to have a scientifically sound and widely accepted measure. The global activity limitation indicator (GALI) is the only internationally agreed indicator of participation restriction, at least in Europe. Still, does the GALI capture the multiple aspects of social participation and their relevance? To answer this question, we use data from the European Health and Social Integration Survey in Spain. We find that GALI adequately reflects the main aspects of participation and is sensitive to the unequal relevance for younger versus older adults. These findings add to the credibility of the GALI as a valid measure of participation restriction in the population.

## Introduction

Summary measures of population health that combine life expectancy and global health measures in a single indicator, i.e. healthy life expectancy, are essential for assessing the performance of public health, clinical medicine, and that of other sectors, such as education and employment, which also influence health [1, 2]. Madans and Weeks [1] have proposed a hierarchical framework for these measures that places healthy life expectancy based on activity limitations/participation restrictions (PR) at the top, followed by those based on functional limitations and perceived health. For global measures of functional limitations and perceived health, there are “definitive” candidates: the six items of the Washington Group on Disability Statistics and Self-Rated Health (SRH); however, for PR, there is not [1, 3]. PR’s complex and all-encompassing nature makes it challenging to achieve a broadly agreed and internationally harmonised summary measure [1, 4].

Verbrugge [5] established three criteria that a PR measure should satisfy: refer to health-related dysfunctions, be strongly associated with participation domains and not be redundant with other global health measures such as SRH. The Euro-REVES 2 project and its prolongation, The European Health and Life Expectancy Information System (EHLEIS) project, consider that the measure should also have an external normative comparison, i.e. refer to things people usually do [3, 6]. Accordingly, the global activity limitation indicator (GALI) was created to measure PR (despite its name, GALI is intended as a measure of PR) [6]. GALI is the only internationally harmonised and agreed-upon PR measure available, but its use is almost exclusively restricted to the EU [7]. It consists of a single item that asks: “For at least the past 6 months, to what extent have you been limited because of a health problem in activities people usually do?”. The experience gathered with GALI is doubly relevant: per se, and as a reference for developing a new, more internationally agreed global PR measure [3].

To what extent does GALI meet favourable evidence on its adequacy to the four criteria mentioned above? First, GALI has been consistently associated with the antecedent health variables for PR development: morbidities, impairments, and functional limitations [8–11]. Therefore, it can be inferred that the reference in GALI wording to limitations linked to health problems works well. Secondly, GALI has been consistently and strongly associated with two PR measures: domestic life and self-care, and once with work restrictions [12]. Although the reference in GALI wording to participation domains is non-specific (“activities people usually do”), the above evidence suggests that the formula may be effective. However, it is unknown whether GALI is associated with measures of other important participation domains. According to two systematic reviews of participation measurement instruments [13, 14], the relevant domains of participation in the International Classification of Functioning Disability and Health (ICF) parlance are mobility, domestic life, self-care, role (studies, work), social relations, civic, social, and community life. Third, GALI has so far shown its added value concerning SRH: it is a complementary predictor of mortality to SRH [15] and reflects PR more strongly than SRH in self-care, domestic life, and work domains [12]. Comparing the performance of GALI with that of SRH facilitates interpreting the value of GALI as a global measure of health; it is, therefore, useful to continue comparing both measures. Fourth, very little is known about the normative performance of GALI: two international studies presented contradictory results on the consistency of GALI performance across countries [8, 10], and one national-level study showed greater homogeneity of GALI across gender than age in adults under 65 [12]. A priori, age (older versus younger adults), is the primary source of heterogeneity in a global PR question [16].

The main objective of this study was (i) to examine whether GALI has a broad coverage of participation domains: education and training, work, mobility, community life and leisure activities, domestic life, and self-care; in addition, we estimated the relative importance of these domains. We also (ii) analysed GALI response homogeneity by age, particularly among adults aged 65 and over versus adults under 65 and gender. Finally, we (iii) compared the performance of GALI versus SRH as global measures of PR. We used data from the European Health and Social Integration Survey (EHSIS) in Spain (EHSIS-S) and selected adults aged 18 and over to answer these objectives.

## Methods

### Data source

The 2012 EHSIS is a cross-national disability survey in 28 European countries among people aged 15 and over living in private households. The EHSIS, without a periodic basis, was inspired by the biopsychosocial model of disability of the ICF but focuses on environmental factors and participation restrictions. The questionnaire followed a procedure to harmonise the language versions based on an original English version. The EHSIS-S, through a multi-stage probabilistic sample design, surveyed 14,600 people, with a response rate of 76.4%. Data collection occurred between July 2012 and April 2013 and was carried out by computer-assisted telephone Interviewing or computer-assisted web Interviewing according to the respondent's choice. The data represent the non-institutionalised Spanish population. Detailed information on the methodology and the quality of the survey can be found on the Spanish National Statistics Institute website [17]. For this research, we select persons aged 18 and over.

### Measures

#### Predictors

We selected three demographic predictors as control variables: age (18–34, 35–44, 45–54, 55–64, 65–74, and 75+), gender, and educational attainment (primary, compulsory secondary, secondary, and university); and six PR predictors: mobility, leisure and community activities, work, education and training, domestic life, and self-care. To measure PR in these domains, except for domestic life and self-care, the EHSIS asked whether there were any impediments to the desired performance (“Is there anything that prevents you from doing… that you want to do?”) of an extensive list including health and functioning (seeing, hearing, concentrating, and moving around) impediments. We classified as PR in the activity/role if health or functioning impediments were among the answers and not PR in all other cases. Besides, the EHSIS used the standard procedure followed in health and disability surveys to measure domestic life and self-care restrictions. Accordingly, we classified as PR in domestic life if the respondent had difficulty, attributed to health or functioning reasons, in at least one of the six activities examined (preparing meals, using the telephone, shopping, managing the medication, housework, and taking care of finances and everyday administrative tasks), and as not PR in all other cases. Finally, we classified as PR in self-care if the respondent had some difficulty in at least one of the five activities surveyed (feeding oneself, getting in and out of bed or chair, dressing and undressing, using the toilet, and bathing or taking a shower) and as not PR in the remaining cases.

#### Washington group on disability statistics short set (WGSS)

The WGSS was designed to identify the population at risk of restricted participation using internationally comparable data [18]. It comprises six items on functioning in core domains: seeing, hearing, walking or climbing steps, cognition, self-care, and communication. Each item has four response options: “no, no difficulty”, “yes, some difficult”, “yes, a lot of difficulty” and “cannot do at all”. The scoring recommended is a binary classification: with a disability, if one or more items, one of the answers is “a lot of difficulty” or “cannot do it at all”, and without disability in the other cases. Although the EHSIS did not include two of the six WGSS items (self-care and walking/stair climbing), it did include sufficient information to construct scores for these two missing items (from five self-care items and two walking/stair climbing items). WGSS scores were used only to describe the level of disability in the sample.

#### Response variables

GALI was the primary outcome variable. GALI is a single-item indicator whose question and response scale are: “For at least the last 6 months, to what extent have you been limited because of a health problem, in activities people usually do? Would you say you have been severely limited, limited but not severely, or not limited at all?” Since the category severely limited is rare in younger age groups, we decided to collapse this category with the category “limited but not severely”; thus, GALI was analysed as a binary variable: not limited vs limited. To measure SRH, the secondary response variable, the EHSIS used the WHO version (How is your health in general? Is it very good, good, fair, bad, or very bad?). Analogous to GALI, it was also analysed as a binary variable: good health (very good/good health) vs fair/bad health (fair, bad, very bad).

### Data analysis

All variables were categorical and are described using frequencies (unweighted data) and percentages (weighted data). To examine whether GALI is a comprehensive measure of participation domains (main objective), we estimated separate logistic regression models with each PR predictor (domain), adjusting for the three demographic variables. We then sequentially estimated the interaction of each domain with age and gender on GALI (second objective), using the chi-square likelihood ratio test to examine the statistical significance of the interaction terms. Then, we calculated (and plotted) the probabilities predicted by these models, according to Muller and MacLehose [19] procedure, to interpret the interactions (with these probabilities, prevalence ratios can be estimated, which lack the non-collapsibility concerns of odds ratios [20]). Based on the results of the interactions, we further analysed the associations between PR predictors and GALI in two age strata, under 65 and over 64. To determine the relative importance of predictors on the response to GALI (also main objective), we used the Shapley value decomposition (SVD) from logistic regression models, including all predictors simultaneously. It is convenient to distinguish the two types of analyses performed to fulfil the main objective. While the relative importance of a predictor is dependent on the strength of its association with the response, its variability, and the set of predictors included (PR and demographic predictors) the strength of the predictor’s association (adjusted for demographic variables) depends only on its (unstandardised) effect on the outcome [21]. SVD is a reliable variance decomposition technique (in this study, the variance according to McFadden R^2^) to determine the relative importance of predictors, even in situations of high multicollinearity [22]. We use 1000 bootstrapped samples to estimate the CIs of the Shapley values. Due to the unequal relevance of some domains between older and younger adults, relative importance analyses were conducted only stratified into two groups: under 65 and over 64. To examine the performance of SRH as a response variable (third objective), we employed the same analysis strategy as for GALI. Stata 11 software was employed to estimate the logistic regression models and Python software to calculate Shapley values; in both instances, we incorporated the scaled sampling weights—based on sample design and calibration techniques to correct non-response bias and improve the precision of the estimates.

## Results

The initial sample size was 14,300 people aged 18 and over. Of these, 642 (4.5%) had some missing values on the selected variables and were removed from the analysis sample. Work and domestic life were the variables with the most missing responses (1.6% and 1.5%, respectively); the rest had missing values of less than 0.8%. The analysis sample consisted of the 13,658 people who submitted complete data on all variables. Table 1 describes the characteristics of the whole sample, the subsample of under 65 and the subsample of over 64. Briefly, 27.92% of the whole sample reported limitations according to GALI (21.76% of under 65 years and 52.36% of over 64); for SRH (fair/bad health), the values were, respectively, 28.42%, 20.55%, and 59.61%. As for the PR predictors, the values ranged from 12.76% for domestic life to 3.72% for education and training. PR in self-care, domestic life, mobility, and social and leisure activities were much higher (four to six times more) in adults over 64 than in adults under 65; however, the percentages of PR in education and training and work were similar in both subsamples.

**Table 1:** Characteristics of participants, overall and by age

Characteristics	Total sample, aged 18+ years, *N* = 13,658		Aged 18–64 years, *N* = 10,287		Aged 65+ years, N = 3371	
	*N*	%	*N*	%	*N*	%
Age						
Mean, (SD): 45, (13.2)						
18–34	2480	26.23	2480	32.84		
35–44	2942	21.07	2942	26.38		
45–54	2741	18.58	2741	23.27		
55–64	2124	13.98	2124	17.50		
65–74	1700	10.71			1700	53.18
75+	1671	9.43			1671	46.82
Sex						
Male	6141	48.4	4837	49.97	1304	42.18
Female	7517	51.6	5450	50.03	2067	57.82
Educational attainment						
Primary	2760	19.49	1110	11.49	1650	51.24
Compulsory secondary	4548	32.5	3431	32.97	1117	30.66
Higher secondary	2657	21.02	2381	24.26	276	8.17
University studies	3693	26.98	3365	31.28	328	9.93
Washington short set						
No disabled	12,196	90.21	9798	95.44	2398	69.47
Disabled	1462	9.79	489	4.55	973	30.53
Work						
No restrictions	12,680	93.05	9499	92.97	3146	93.33
Restrictions	978	6.95	788	7.02	225	6.67
Education and training						
No restrictions	13,143	96.23	9877	96.22	3255	96.56
Restrictions	515	3.72	410	3.78	116	3.44
Mobility						
No restrictions	12,199	90.21	9690	94.19	2539	74.43
Restrictions	1459	9.79	627	5.81	832	25.57
Community and leisure						
No restrictions	11,955	88.62	9505	92.97	2450	71.37
Restrictions	1703	11.38	782	7.03	832	28.63
Domestic life						
No restrictions	11,689	87.24	9496	93.06	2193	64.15
Restrictions	1969	12.76	791	6.94	1178	35.85
Self-care						
No restrictions	12,146	90.29	9785	95.64	2361	69.04
Restrictions	1512	9.71	502	4.36	1010	30.96
GALI						
Not limited	9574	72.08	7929	78.24	1645	47.64
Limited	4084	27.92	2358	21.76	1726	52.36
SRH						
Good	9423	71.58	8015	79.45	1408	40.39
Fair/bad	4235	28.42	2272	20.55	1963	59.61

### Associations between predictors and GALI and SRH

#### All sample: adults 18 and over

PR predictors, adjusted for demographic variables, were strongly associated with GALI and with SRH. Most associations were heterogeneous across age but not gender (except for the associations of domestic life with GALI (chi-square difference = 7, df = 1, *p* < 0.01) and SRH (chi-square difference = 4.1, df = 1, *p* < 0.05), which were slightly higher in males). The probabilities predicted by the models with interactions of age and PR predictors on GALI and SRH showed a trend towards a less strong association between the predictors and the two response variables with increasing age, more pronounced from 55 or 65 onwards (Supplementary Figs. S1, S2).

#### Adults under 65

PR predictors were strongly associated with GALI, with adjusted ORs ranging from 29.47 (95% CI 21.97, 39.52) for work to 20.48 (95% CI 15.65, 26.80) for community and leisure activities. Table 2 presents the results for GALI and SRH. The associations between PR predictors and SRH were strong but lower than those observed with GALI, with adjusted ORs ranging between 23.25 (95% CI 17.13, 31.55) for work and 15.75 (95% CI 12.22, 20.31) for domestic life. However, age and educational attainment were more strongly associated with SRH than with GALI.

**Table 2:** Associations, raw and adjusted for demographics, of predictors with GALI and SRH

Predictors	Unadjusted odds ratio (95% CI)		Adjusted odds ratio (95% CI)	
	GALI = limited	SRH = fair/bad	GALI = limited	SRH = fair/bad
Education and training				
No restrictions	Ref.	Ref.	Ref.	Ref.
Restrictions	29.80 (19.21–46.21)	25.09 (16.59–37.96)	22.31 (14.46–34.42)	16.21 (10.60–24.80)
Work				
No restrictions	Ref.	Ref.	Ref.	Ref.
Restrictions	36.02 (26.88–48.27)	32.78 (24.49–43.88)	29.47 (21.97–39.52)	23.25 (17.13–31.55)
Mobility				
No restrictions	Ref.	Ref.	Ref.	Ref.
Restrictions	32.65 (23.29–45.78)	24.08 (17.86–32.46)	27.95 (19.97–39.12)	21.72 (15.95–29.57)
Community and leisure				
No restrictions	Ref.	Ref.	Ref.	Ref.
Restrictions	24.37 (18.64–31.87)	21.72 (17.04–27.68)	20.48 (15.65–26.80)	17.67 (13.65–22.89)
Domestic life				
No restrictions	Ref.	Ref.	Ref.	Ref.
Restrictions	30.46 (23.26–39.89)	22.05 (17.43–27.89)	24.34 (18.53–31.97)	15.75 (12.22–20.31)
Self-care				
No restrictions	Ref.	Ref.	Ref.	Ref.
Restrictions	29.33 (21.06–40.85)	23.87 (17.49–32.57)	22.47 (16.05–31.46)	16.83 (12.05–23.51)
Age				
18–34	Ref.	Ref.	Ref.	Ref.
35–44	1.30 (1.09–1.56)	2.25 (1.81–2.78)	1.32 (1.10–1.58)	2.33 (1.87–2.89)
45–54	1.84 (1.55–2.18)	3.95 (3.23–4.83)	1.75 (1.47–2.08)	3.66 (2.99–4.89)
55–64	2.94 (2.47–3.50)	7.69 (6.28–9.42)	2.54 (2.12–3.04)	6.00 (4.88–7.39)
Gender				
Male	Ref.	Ref.	Ref.	Ref.
Female	1.37 (1.22–1.54)	1.46 (1.29–1.64)	1.38 (1.22–1.55)	1.51 (1.33–1.71)
Education attainment				
University	Ref.	Ref.	Ref.	Ref.
Higher secondary	1.40 (1.18–1.65)	1.89 (1.56–2.30)	1.41 (1.19–1.67)	1.98 (1.62–2.42)
Comp. secondary	1.69 (1.45–1.95)	3.43 (2.90–4.04)	1.52 (1.31–1.77)	2.97 (2.51–3.51)
Primary	2.96 (2.41–3.56)	6.90 (5.61–8.49)	2.38 (1.93–2.92)	5.20 (4.18–6.47)

*N* = 10,287, 18–64 old years

#### Adults over 64

Table 3 presents the results for GALI and SRH. Adjusted ORs between each PR predictor and GALI were strong but lower than those found in the under-65 subsample. For example, the adjusted OR between domestic life and GALI was 13.33 (95% CI 10.42, 17.03) in adults over 64 and 24.34 (95% CI 18.53, 31.97) in adults under 65. Parallel results were observed with SRH (e.g. the adjusted OR between domestic life and SRH was 7.39 (95% CI 5.80, 9.42) vs 15.75 (95% CI 12.22, 20.31), but overall, the associations were less strong than with GALI. Finally, educational attainment continued to be more highly associated with SRH than with GALI, but age was not.

**Table 3:** Associations, raw and adjusted for demographics, of predictors with GALI and SRH

Predictors	Unadjusted odds ratio (95% CI)		Adjusted odds ratio (95% CI)	
	GALI = limited	SRH = fair/bad	GALI = limited	SRH = fair/bad
Studies				
No restrictions	Ref.	Ref.	Ref.	Ref.
Restrictions	7.87 (3.98–15.59)	10.62 (4.47–25.23)	9.39 (4.74–19.03)	11.71 (4.74–28.91)
Work				
No restrictions	Ref.	Ref.	Ref.	Ref.
Restrictions	10.44(5.57–19.56)	11.67 (6.15–22.16)	15.33 (7.88–29.82)	15.51 (7.79–30.89)
Mobility				
No restrictions	Ref.	Ref.	Ref.	Ref.
Restrictions	23.91 (16.82–33.99)	15.34 (11.23–20.94)	19.57 (13.76–27.85)	12.26 (8.94–16.80)
Community and leisure				
No restrictions	Ref.	Ref.	Ref.	Ref.
Restrictions	17.32 (12.97–23.12)	12.01 (9.07–15.89)	14.50 (10.82–19.43)	10.28 (7.74–13.64)
Domestic life				
No restrictions	Ref.	Ref.	Ref.	Ref.
Restrictions	15.48 (12.22–19.61)	8.95 (7.13.11.24)	13.33 (10.42–17.03)	7.39 (5.80–9.42)
Self-care				
No restrictions	Ref.	Ref.	Ref.	Ref.
Restrictions	16.45 (12.73–21.42)	9.31 (7.30–11.87)	13.62 (10.49–17.67)	7.72 (6.0–9.91)
Age				
65–74	Ref.	Ref.	Ref.	Ref.
75+	2.54 (2.15–3.01)	2.05 (1.73–2.43)	1.98 (1.72–2.29)	1.52 (1.31–1.76)
Gender				
Male	Ref.	Ref.	Ref.	Ref.
Female	2.01 (1.70–2.38)	1.74 (1.46–2.06)	1.76 (1.53–2.04)	1.52 (1.31–1.76)
Education attainment				
University	Ref.	Ref.	Ref.	Ref.
Higher secondary	0.99 (0.67–1.48)	1.17 (0.79–1.75)	0.95 (0.68–1.34)	1.20 (0.86–1.69)
Comp. secondary	1.28 (0.94–1.48)	2.18 (1.60–2.97)	1.01 (0.78–1.31)	1.93 (1.49–2.51)
Primary	2.12 (1.58–2.84)	4.69 (3.46–6.35)	1.59 (1.24–2.05)	3.70 (2.86–4.79)

N = 3371, 65+ old years

### The relative importance of predictors

**Fig. 1:**
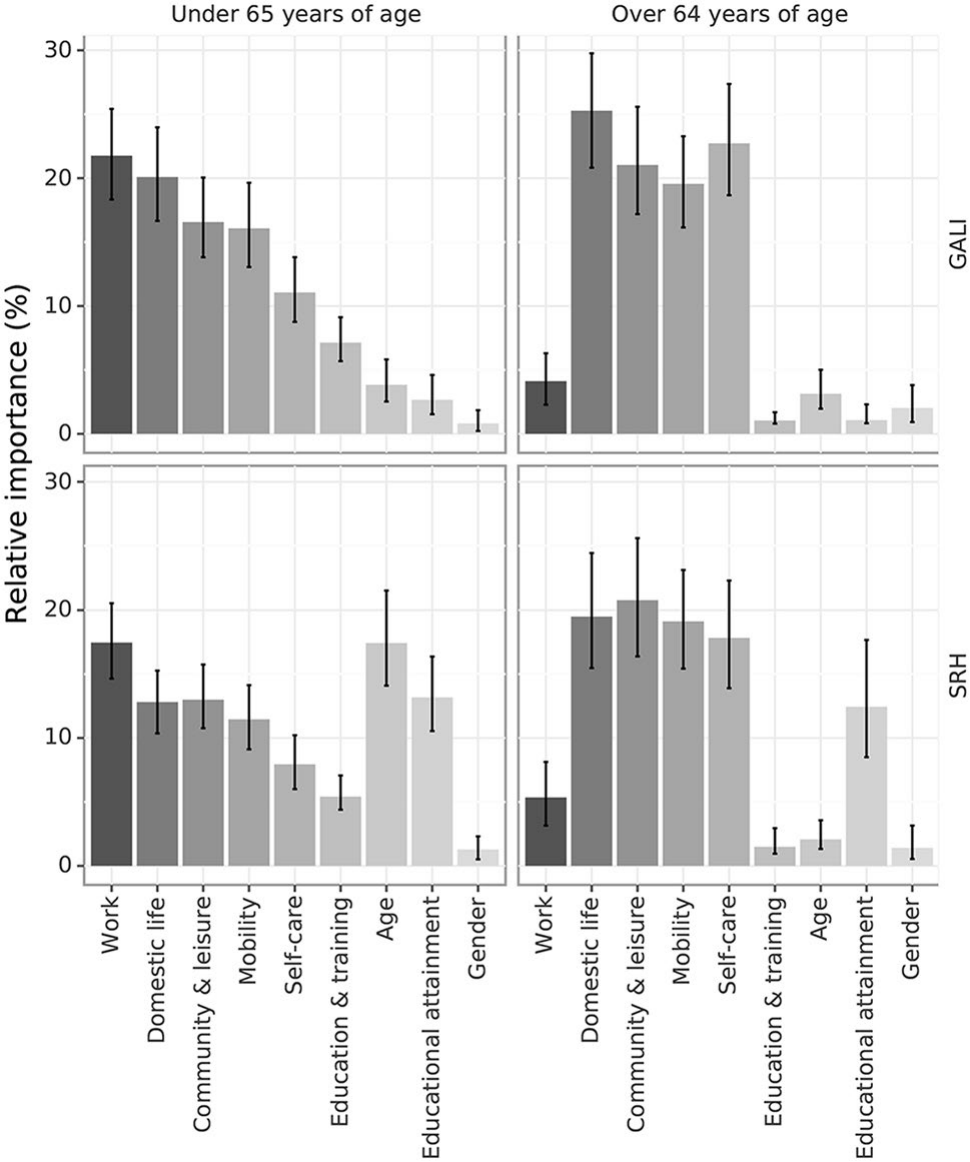
Relative importance of predictors on GALI and SRH in older and younger adults

Figure 1 represents the relative importance of predictors, according to the SVD, in adults under 65 and Fig. 1 in adults over 64. Supplementary Table S1 shows results from the respective logistic regression models on which the SVD was based. For the response to GALI, in adults under 65 (McFadden *R*^2^ = 0.222), the relative importance of PR predictors ranged from 21.78% (work) to 7.12% (education and training), and the demographic variables had minor importance (7.3%). In the case of SRH (McFadden *R*^2^ = 0.273), PR predictors were less important and ranged from 17.46% for work to 5.44% for education and training; age was the second most important predictor (17.43%), and educational attainment was the fourth (13.2%).

In adults over 64, for GALI (McFadden *R*^2^ = 0.321), domestic life was the most important predictor, and education and training and work were by far the two least important predictors. The demographic predictors had joint importance of 6%. For SRH (McFadden *R*^2^ = 0.236), education and training and work were also the two most minor important predictors, and the other four predictors ranged from 20.79% for community and leisure activities to 17.81% for self-care. The relative importance of age decreased and was lower than in GALI, but educational attainment was still relatively important (12.67%).

## Discussion

In this study using data from the EHSIS, GALI reflected the domains of participation and their relative importance well: it was strongly associated with each PR predictor, and the relative importance of these differed among adults over 64 versus under 65. The association between PR predictors and GALI was largely homogeneous across gender but was heterogeneous across age: stronger in adults under 65 than in adults over  64. The results with SRH were parallel to those of GALI, but the association of SRH with PR predictors was lowest, and age appeared to introduce more heterogeneity in the response to SRH than to GALI.

GALI item uses the expression *in activities people usually do* to investigate whether people experience global PR. Is this formula effective in achieving a comprehensive measure of participation domains? Our results indicated that it is. Thus, GALI was strongly associated in models adjusted for demographics, with each PR predictor examined: self-care and domestic life [15], work [12], education and training, mobility, and community life and leisure activities. Furthermore, the relative importance of all domains, as examined by SVD, was remarkable and varied in adults under 65 versus over 64, in line with the expected relevance of domains for each age group [23, 24]. Thus, while work (and then domestic life) was the most important domain among adults under 65, among those aged 65 and over, domestic life (and then self-care) was the most important domain, and work was scarcely important.

In addition to GALI, other standardised items ask about global PR. For example, one of the five items of the EQ-5D refers to *usual activities*, but, unlike GALI, the activities concerned (housework, leisure, work, and family) are listed. Gamst-Klaussen and Lamu [25] examined with SVD the relative importance of the four domains intended by the *usual activities* item. All four domains were important, although housework activities were the most important. The authors concluded that the item does indeed measure what it is intended to measure. It seems, therefore, that both strategies—GALI: *activities people usually do*, EQ-5D: *usual activities* and a list of intended activities—are adequate for their respective purposes. However, applying the EQ-5D strategy to a measure with a more general purpose such as GALI would entail a significant loss of conciseness (the item statement should reference domains such as work, school, self-care, social relations, housework, mobility, community life, and leisure pursuits), which is a critical technical feature for a summary measure [3]. Also, as noted in the previous paragraph, some domains may have unequal relevance depending on age [4]. Without reference to any specific participation domain, the GALI statement appears to capture both multiple domains of participation and their unequal relevance linked to age. As proposed by Verbrugge [5], a global eclectic (i.e. not referring to any specific domain) indicator on activities is helpful for all adult ages and avoids perpetuating bias.

In this study, GALI was homogeneously associated with PR measures across gender but not across age: in adults under 65, the association was higher than in adults over  64. The importance of age in global health judgements is linked to a higher frequency of health and functioning problems [26]. One consequence is that older people may modify their frame of reference on health to adapt it to the normality of old age [27], which would explain why, in older people, global health assessments (SRH) are less strongly associated with specific health or functioning measures [28]. The observed lower association of GALI with specific measures of PR in older adults could imply that older adults change (in parallel to what happens with SRH) their standards of good functioning.

Compared to GALI, SRH performance was worse as a global measure of functioning as it was less strongly associated with predictors of PR in both adults under 65 and over 64. In contrast, SRH was more strongly associated than GALI with two demographic variables: age among adults under 65 and educational attainment in both age groups. This greater effect of educational attainment on SRH than on GALI may be relevant in research on health inequalities. Indeed, in a multinational study on health inequalities in Europe, SRH also presented a larger gradient by educational attainment than GALI [29]. Finally, the association of SRH with PR predictors was, as with GALI, largely homogeneous by gender and heterogeneous by age [27]. This heterogeneity might be greater with SRH than with GALI because the effect of age in older adults seems to increase for GALI, but not for SRH. Consequently, in older people, the validity of SRH as a proxy measure of health would be relatively lower than GALI’s as a measure of function.

### Strengths and limitations

We highlight some strengths of this research. It is the first time, to our knowledge, to examine whether GALI is a comprehensive measure of the main domains of participation, and it is also original in using SVD to determine the relative importance of each PR domain. Furthermore, the analysis sample was nationally representative (Spain), large, and had high participation rates.

Some limitations are the following. First, while the measures used for self-care and domestic life restrictions are common to those generally employed in health and disability surveys, the rest of the PR measures were created ad hoc for the EHSIS and had uncertain validity. Second, the strategy for measuring PR in GALI differs from that of the specific PR measures (except for self-care and, partially, domestic life): GALI asks only about health-related restrictions, while the specific measures ask about restrictions in general and then identify health-related restrictions. This second strategy, as opposed to the first, may underestimate the prevalence of RP in these domains [30]. Moreover, the predictors thus measured would have decreased variance and, therefore, their relative importance would also be underestimated. Third, our results are only statistically generalisable to the Spanish population. However, evidence suggests that these results have a wider-reaching: two European cross-national studies that examined the associations of self-care, domestic life, and functional limitations with GALI showed that the values for Spain were very close to the general values [8, 10].

## Conclusions

GALI item was conceived as a global measure of PR. To this end, GALI asks about limitations in activities that people usually do. Our findings indicate that this formula achieves a reflective measure of restrictions in multiple participation domains and their relative importance. Furthermore, GALI performs homogeneously between men and women, but not between older and younger adults, probably because older people lower their good functioning standards. To a lesser extent, this phenomenon is similar to that observed with SRH and the change in good health standards.

## Supplementary Information

Below is the link to the electronic supplementary material.

Supplementary file1 (PDF 309 KB)

Supplementary file2 (PDF 323 KB)

Supplementary file3 (PDF 41 KB)

## Data Availability

The study’s raw data are available at the Spanish National Statistics Institute (INE) website https://www.ine.es/dyngs/INEbase/en/operacion.htm?c=Estadistica_C&cid=1254736176987&menu=resultados&idp=1254735573175. The curated data that support the findings of this study are available from the corresponding author, [julio.cabrero@ua.es], upon reasonable request.
