# Supplementary material for: Does the global activity limitation indicator measure participation restriction? Data from the European Health and Social Integration Survey in Spain
Source: Qual Life Res. 2021 Dec 9;31(5):1335–44. doi: 10.1007/s11136-021-03057-z (PMC9023392; doi:10.1007/s11136-021-03057-z)
Supplement: Supplementary file 1 — Supplementary file1 (PDF 309 KB) [file 11136_2021_3057_MOESM1_ESM.pdf]

Online Resource 1 Probabilities of response to GALI predicted by the interactions between age and participation restriction (PR) domains. Probabilities have been calculated from logistic regression models including demographic covariates, the participation restriction domain, and the interaction term

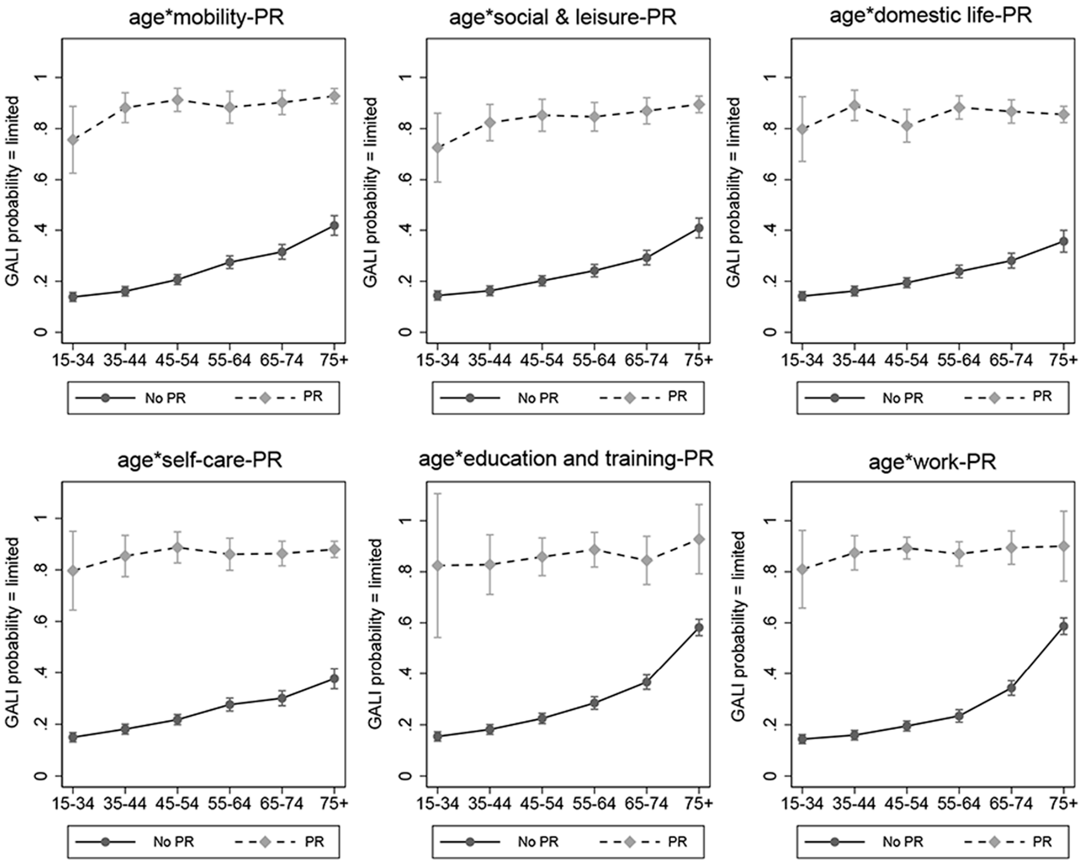

Article title: Does the Global Activity Limitation Indicator measure participation restriction? Data from the European Health and Social Integration Survey in Spain.

Journal name: Quality of Life Research.

Author names: Julio Cabrero-García[a], Juan Ramón Rico-Juan[b], Antonio Oliver-Roig[c].

Affiliation: [a, c]Department of Nursing, University of Alicante. [b]Department of Software and Computing Systems, University of Alicante.

Corresponding author: Julio Cabrero-García. E-mail: julio.cabrero@ua.es
