# Supplementary material for: Does the global activity limitation indicator measure participation restriction? Data from the European Health and Social Integration Survey in Spain
Source: Qual Life Res. 2021 Dec 9;31(5):1335–44. doi: 10.1007/s11136-021-03057-z (PMC9023392; doi:10.1007/s11136-021-03057-z)
Supplement: Supplementary file 3 — Supplementary file3 (PDF 41 KB) [file 11136_2021_3057_MOESM3_ESM.pdf]

Online Resource 3 Associations of all predictors, simultaneously adjusted, with GALI and SRH

	Aged 18-64 years, N = 10287		Aged 65+ years, N = 3371	
	Fully adjusted odds ratio (95% CI)		Fully adjusted odds ratio (95% CI)	
Predictors	GALI = limited	SRH= fair/bad	GALI = limited	SRH= fair/bad
Education & training				
No restrictions	Ref.	Ref.	Ref.	Ref.
Restrictions	0.87 (0.35-2.12)	0.63 (0.30-1.35)	0.45 (0.16-1.24)	1.06 (0.33-3.39)
Work				
No restrictions	Ref.	Ref.	Ref.	Ref.
Restrictions	5.54 (3.79-8.11)	4.98 (3.43-7.25)	3.31 (1.50-7.29)	3.73 (1.69-8.24)
Mobility				
No restrictions	Ref.	Ref.	Ref.	Ref.
Restrictions	4.49 (2.76-7.31)	3.78 (2.43-5.89)	3.02 (1.94-4.70)	2.64 (1.78-3.92)
Community & leisure				
No restrictions	Ref.	Ref.	Ref.	Ref.
Restrictions	3.03 (2.08-4.42)	2.97 (2.04-4.32)	3.51 (2.45-5.03)	2.96 (2.12-4.14)
Domestic life				
No restrictions	Ref.	Ref.	Ref.	Ref.
Restrictions	4.49 (3.13-6.46)	2.64 (1.86-3.73)	3.39 (2.53-4.54)	2.04 (1.52-2.73)
Self-care				
No restrictions	Ref.	Ref.	Ref.	Ref.
Restrictions	3.67 (2.30-5.88)	2.62 (1.61-4.26)	3.54 (2.58-4.84)	2.25 (1.67-3.03)
Age				
18-34	Ref.	Ref.	-	-
35-44	1.17 (0.92-1.36)	2.18 (1.69-2.80)	-	-
45-54	1.36 (1.12-1.66)	3.34 (2.64-4.23)	-	-
55-64	1.55 (1.25-1.91)	4.64 (3.63-5.92)	-	-
65-74	-	-	Ref.	Ref.
75+	-	-	1.18 (0.95-1.48)	0.96 (0.78-1-18)
Gender				
Male	Ref.	Ref.	Ref.	Ref.
Female	1.21 (1.05-1.39)	1.39 (1.20-1.60)	1.30 (1.05-1.61)	1.13 (0.93-1.38)
Educ. attainment				
University	Ref.	Ref.	Ref.	Ref.
Higher secondary	1.27 (1.05-1.53)	1.89 (1.53-2.35)	0.91 (0.59-1.41)	1.16 (0.74-1.83)
Comp. secondary	1.15 (0.97-1.38)	2.65 (2.19-3.20)	0.85 (0.61-1.18)	1.90 (1.33-2.70)
Primary	1.54 (1.19-1.99)	4.20 (5.27-5.40)	0.85 (0.60-1.18)	3.13 (2.20-4.45)

Article title: Does the Global Activity Limitation Indicator measure participation restriction? Data from the European Health and Social Integration Survey in Spain.

Journal name: Quality of Life Research.

Author names: Julio Cabrero-García[a], Juan Ramón Rico-Juan[b], Antonio Oliver-Roig[c].

Affiliation: [a, c]Department of Nursing, University of Alicante. [b]Department of Software and Computing Systems, University of Alicante.

Corresponding author: Julio Cabrero-García. E-mail: julio.cabrero@ua.es
